# Validating the Modified Drug Adherence Work-Up (M-DRAW) Tool to Identify and Address Barriers to Medication Adherence

**DOI:** 10.3390/pharmacy5030052

**Published:** 2017-09-08

**Authors:** Sun Lee, Yuna H. Bae, Marcia Worley, Anandi Law

**Affiliations:** 1 College of Pharmacy, Western University of Health Sciences, Pomona, CA 91766, USA; slee22@westernu.edu; 2 School of Pharmacy, University of Southern California, Los Angeles, CA 90089, USA; jaehee616@gmail.com; 3 College of Pharmacy, Ohio State University, Columbus, OH 43210, USA; worley.18@osu.edu

**Keywords:** medication adherence checklist, identifying barriers to adherence, Modified Drug Adherence Work-up Tool, addressing barriers to medication adherence

## Abstract

Barriers to medication adherence stem from multiple factors. An effective and convenient tool is needed to identify these barriers so that clinicians can provide a tailored, patient-centered consultation with patients. The Modified Drug Adherence Work-up Tool (M-DRAW) was developed as a 13-item checklist questionnaire to identify barriers to medication adherence. The response scale was a 4-point Likert scale of frequency of occurrence (1 = never to 4 = often). The checklist was accompanied by a GUIDE that provided corresponding motivational interview-based intervention strategies for each identified barrier. The current pilot study examined the psychometric properties of the M-DRAW checklist (reliability, responsiveness and discriminant validity) in patients taking one or more prescription medication(s) for chronic conditions. A cross-sectional sample of 26 patients was recruited between December 2015 and March 2016 at an academic medical center pharmacy in Southern California. A priming question that assessed self-reported adherence was used to separate participants into the control group of 17 “adherers” (65.4%), and into the intervention group of nine “unintentional and intentional non-adherers” (34.6%). Comparable baseline characteristics were observed between the two groups. The M-DRAW checklist showed acceptable reliability (13 item; alpha = 0.74) for identifying factors and barriers leading to medication non-adherence. Discriminant validity of the tool and the priming question was established by the four-fold number of barriers to adherence identified within the self-selected intervention group compared to the control group (4.4 versus 1.2 barriers, *p* < 0.05). The current study did not investigate construct validity due to small sample size and challenges on follow-up with patients. Future testing of the tool will include construct validation.

## 1. Introduction

Poor medication adherence has been shown to contribute to approximately 33–69% of medication-related hospital admissions in the United States [1,2]. It is estimated that the avoidable health care costs related to medication non-adherence are approximately $100–300 billion a year [1]. Medication non-adherence, defined as patients not taking medications as directed, is either intentional or unintentional. Typically, intentional non-adherence is associated with patients’ motivation and beliefs, and unintentional non-adherence is related to patients’ skills or ability to take medications [2]. Commonly cited factors related to unintentional non-adherence are complex therapy regimens, language barriers, difficulty accessing medications and cognitive problems. Reasons for intentional non-adherence include lack of perceived benefit of the medication, fear of side effects and lack of trust in healthcare providers [3]. For these reasons, it is essential for healthcare providers to understand the drivers of medication non-adherence in each patient in order to provide tailored therapy plans for patients.

Several studies have looked at methods to screen patients who may be at risk for non-adherence. Notably, the eight-item Morisky Medication Adherence Scale [4], Brief Medication Questionnaire [5] and Adherence Barriers Questionnaires [6] are commonly used to assess patients’ self-reported adherence level. These tools tend to be lengthy and/or fall short of providing guidance on addressing the identified issues that contribute to non-adherence. Doucette et al. developed the Drug Adherence Work-up (DRAW) tool to both identify barriers to adherence and to provide strategies to address the identified problems [7]. However, given that the barriers to medication adherence are multifactorial in nature (patient-related, therapy-related, condition-related, health system-related) [8], there needs to be a more comprehensive and multidimensional tool that can both identify these challenges and address them.

The current research team worked on expanding the DRAW tool with permission from the original tool developers, using mixed methods. A qualitative interview study conducted among patients with one or more chronic health conditions at a local clinic explored the role of patient expectations on secondary medication non-adherence using content analysis. The study identified perceived benefits and barriers, negative attributes to taking medications and external and inherent influences to action about medication adherence. In addition, a review of published models (Table 1) explaining barriers to medication adherence was used to develop the 13-item Modified Drug Adherence Work-up (M-DRAW) tool. Four domains were proposed for the M-DRAW: socioeconomic (1 item), patient-related (3 items), therapy-related (5 items), and condition-related (2 items) (Figure S1), ordered from least-challenging barriers to address (transportation to receive medications) to most-challenging barriers (patient beliefs). In addition, two questions addressed patient characteristics to make up the 13-item M-DRAW tool.

The investigators modified response choices from ‘yes’ or ‘no’ options in the DRAW to a 4-point Likert scale (1 = never, 2 = rarely, 3 = sometimes, or 4 = often). The rationale was that scaled answer choices would help patients reflect on any challenges associated with their true medication-taking behavior that may be missed with dichotomous choices. Similar to the original DRAW tool, the modified version also provided strategies to perform a patient-centered, motivational interviewing (MI)-based intervention. Thus, for each barrier, Guided Strategies for Increasing Adherence (GUIDE) (Figure S1, pages 3–4) that could help healthcare providers tailor interventions to patients were added to the M-DRAW.

The tool was then assessed for face and content validity by a panel of expert clinicians (4), researchers (4) (including one of the original DRAW tool developers), motivational interviewing trainers (2) and lay individuals (3). Based on responses from the panel, some items and the scale were modified. The objective of the current study was to examine the psychometric properties of the M-DRAW tool for internal consistency and responsiveness.

## 2. Materials and Methods

### 2.1. Sample and Design

A prospective, pre-post interview design was used to validate the M-DRAW tool. This study was conducted in an academic medical center pharmacy in Southern California. Eligibility criteria for inclusion in the study consisted of: English-speaking patrons who were 18 years or older, residing in a non-institutional setting and taking one or more prescription medication(s) regularly for any chronic condition(s). Purposive sampling was used to recruit participants by examining active prescription records at the pharmacy. A licensed pharmacist in the academic medical center pharmacy reviewed active prescriptions from the computer-based system. This pharmacist then identified potential patients who met the study inclusion criteria. These potential patients were contacted over the phone. Additionally, walk-in participants were recruited at the pharmacy when they came in to fill their prescription(s).

#### Sample Size

The study was designed to detect a moderate effect size of 0.5 between the control and intervention groups. Sample size was estimated at 64 participants for each group based on a power of 80% and a two-tailed alpha level of 0.05. Assuming the traditional attrition rate of 25%, sample recruitment was aimed at a total 172 participants (86 participants each in the control and intervention groups).

### 2.2. Ethics

Study protocol and tools received exempt approval from the Institutional Review Board at Western University of Health Sciences, Pomona, CA. Eligible and willing participants were informed about the study and provided an informed consent form to sign before beginning the study. Participants were given an information sheet explaining the study procedure and principle investigator’s contact information. All participants were assured that their responses would be confidential and that they could withdraw from the study at any time they wished, without any consequences.

Using the priming question, participants were grouped into control and intervention groups. This self-selection group assignment method was chosen to alleviate ethical concerns that may arise with randomized design where patients who identify themselves as at risk for non-adherence could receive random assignment to the control group, thereby not receiving the needed care to address adherence issues. Instead, by using self-selection to allocate those “committed to adherence” into the control group, intervention strategies could be directed to those uncertain of their medication adherence and thus needing help with it (intervention group).

### 2.3. Data Collection

The study was conducted from December 2015 to March 2016. Each participant was asked to complete (1) a demographic survey consisting of age, gender, ethnicity, annual income, education level, types of chronic health condition(s) and prescription drug coverage (yes or no) (Figure S2); (2) the priming question (refer to Section 2.2.1 for additional information) and M-DRAW tool (Figure S1). Participants were asked to fill out the above-mentioned survey forms (Figures S1 and S2). A licensed pharmacist was available while participants were completing the survey forms. In addition, baseline clinical measures and the average proportion of days covered (PDC) rate as a measure of patient adherence were collected for each participant.

#### 2.3.1. Priming Question

As mentioned before, each participant was asked to indicate their perceived medication-adherence level by answering a single-item question with three choices (Table 2). Based on their response, they were categorized into one of three adherence categories: outright non-adherence (ONA or intentional non-adherers), partial non-adherence (PNA or unintentional non-adherers) and adherers (Table 2).

**Figure 1:**
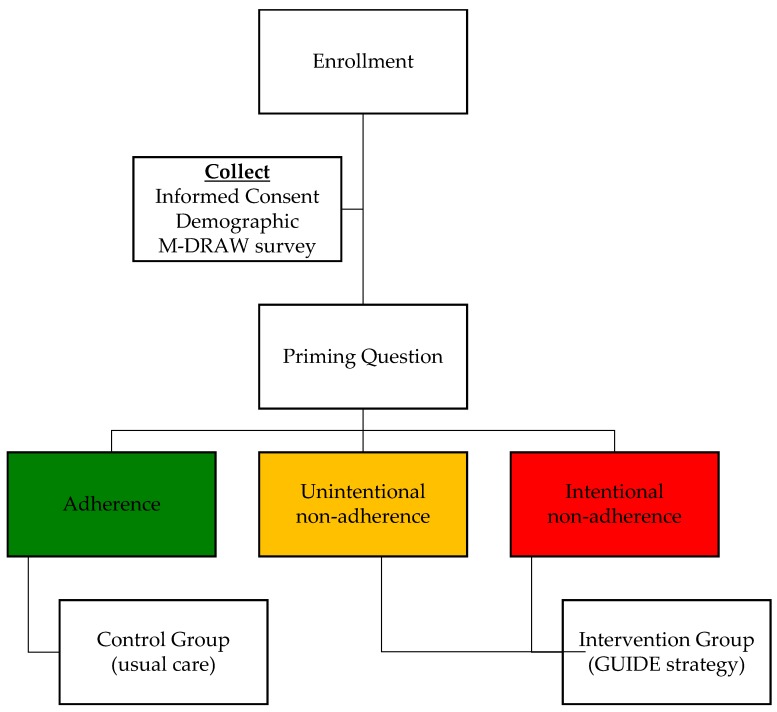
Flow chart of study enrollment and data collection process. M-DRAW, Modified Drug Adherence Work-up (Figure S1, Pages 1–2); GUIDE, Guided Strategies for Increasing Adherence (Figure S1, Pages 3–4).

Participants who chose either (a) or (c) were assigned to the intervention group (received the adherence intervention); and those who chose response (b) served as the control group who would need no adherence-promoting intervention (Figure 1).

#### 2.3.2. Intervention

Once participants completed the priming question and M-DRAW survey (Figure S1, pages 1–2), a student pharmacist, trained on the study protocol, screened responses and assigned the participants to their relevant group. They then reviewed the intervention group responses for any items that were answered either ‘3 = sometimes’ or ‘4 = often’. The intervention group then received the relevant MI-based GUIDE counseling from a licensed pharmacist (also trained on the study protocol) to address each identified barrier. The pharmacist was instructed to check off the box next to the intervention strategies to indicate that the intervention strategy had been discussed with the participant. The control group also completed the surveys, but only received the usual care from the pharmacist.

#### 2.3.3. Proportion of Days Covered (PDC) Rate and Clinical Indicators

To supplement the self-reported adherence rate (measured by using the priming question), average proportion of days covered (PDC) and clinical indicators were collected to compare between the control and intervention groups. Each participant’s average PDC rate was calculated using the medication-filling history dated back to six months prior to the study enrollment. If the filling history was not available because medications were newly initiated, the PDC was not calculated.

Many patients who filled prescriptions at the pharmacy received care from the providers at the medical center clinics. Hence, clinical measures were available to include and evaluate in the study: high blood pressure (systolic and diastolic blood pressure), dyslipidemia (lipid panels) and type 2 diabetes mellitus control (hemoglobin A1C).

#### 2.3.4. Follow-Up

For the post-test, participants were followed-up after three months, either in person or over the phone, to administer the M-DRAW tool by a trained licensed pharmacist or a student pharmacist under the supervision of a trained licensed pharmacist. Upon completion of both baseline and follow-up sessions, patients were given a $10 gift card as gratitude for their time and efforts.

### 2.4. Statistical Analyses

SPSS version 24 (SPSS Inc., Chicago, IL, USA) was used to analyze data. For continuous variables such as age, PDC score and clinical measures, an independent *t*-test was used to compare the control and intervention groups. Chi-squared tests were used to test differences between the intervention and control groups on gender, ethnicity, income, education level, chronic conditions and status of prescription drug coverage via insurance. Responses to M-DRAW tool items were dichotomized and recoded to count the number of barriers (i.e., each item answered ‘1 = never’ or ‘2 = rarely’ was recoded to ‘0’ and each item answered ‘3 = sometimes’ or ‘4 = often’ was recoded to ‘1’). Then, the total number of barriers identified between the groups was compared using the independent *t*-test. Internal consistency was evaluated using Cronbach’s coefficient alpha with an acceptable value of greater than 0.70.

## 3. Results

### 3.1. Response Rate

Of the 54 potential participants screened for eligibility and approached, 26 subjects (48.1%) agreed to enroll in the study. Reasons for non-participation included not meeting study inclusion criteria (*n* = 3), lack of interest (*n* = 12), no time (*n* = 7), or other reasons (*n* = 6). The study had to be stopped a month after baseline data were collected due to lack of clinic personnel to help with data collection. Therefore, only baseline data were analyzed and reported.

### 3.2. Self-Reported Adherence Level

Of the 26 subjects recruited, 17 (65.4%) were assigned to the control group (adherent to the medication) and 9 (34.6%) reported partial adherence (PNA or unintentional non-adherence) and were assigned into the intervention group. No one self-reported outright non-adherence (ONA or intentional non-adherence).

### 3.3. Demographic Characteristics

Baseline characteristics were comparable between the control and intervention groups, except for prescription drug insurance coverage (Table 3). The mean age of participants was 58 ± 8.4 years. About 53.8% were female, 38.5% were white, 61.5% had high school and some college level education and 57.7% earned more than $25,000 annually. Approximately 77.8% answered having prescription drug coverage in the intervention group, compared to 100% in the control group (*p* < 0.05).

### 3.4. Adherence Rate and Clinical Measures

On average, at baseline, the intervention group had four-times greater number of barriers identified (answering each item either ‘sometimes’ or ‘often’) compared to the control group (4.4 ± 2.5 items vs. 1.2 ± 1.3, *p* < 0.05) (Table 4). The PDC rate in the intervention group was lower than the control group; however, the difference was not statistically significant (77.1 ± 21.0 vs. 80.4 ± 10.3, *p* = 0.687). Clinical markers (e.g., hemoglobin A1C, blood pressure and lipid panel) were available in select patients who received recent care at the ambulatory care clinic. The intervention group showed lower A1C (6.4 ± 1.1 vs. 7.8 ± 1.3, *p* > 0.05) and total cholesterol (152 ± 45.3 vs. 193 ± 37.7, *p* > 0.05).

### 3.5. Psychometric Properties

The reliability analysis revealed an overall Cronbach’s alpha of 0.74 (*n* = 26). Due to a small sample size, factor analysis was not performed to confirm the instrument’s construct validity.

### 3.6. Responses to the M-DRAW Checklist

Overall, each item showed a trend of higher average score (more frequent barriers) in the intervention group compared to the control group. Two items (questions 4 and 6) showed a statistically significant difference between the two groups.

## 4. Discussion

The purpose of the current study was to examine the psychometric properties of the Modified Drug Adherence Work-up (M-DRAW) tool for internal consistency and responsiveness. The M-DRAW tool appears to be reliable and somewhat valid. While a larger sample size is needed to confirm construct validity, this initial phase study showed promise on assessing barriers contributing to the medication non-adherence. To the best of our knowledge, this tool is the first of its kind to assess a patient’s self-reported adherence level using a single-item question, called the priming question.

Measuring adherence at the patient level using a single-item question (i.e., priming question) showed good discriminant validity. The priming question categorized patients into those who reported adherence (control group) and those who reported partial non-adherence (PNA, intervention group). There was a non-significant difference in proportion of days covered (PDC) rate between the two groups (*p* > 0.05); however, a greater number of barriers was identified using the M-DRAW tool in the intervention group compared to the control group (Table 5). This finding suggests that the priming question could be utilized to identify patients who have issues with non-adherence. Interestingly, of the 26 subjects recruited, none was categorized as outright (intentional) non-adherence (ONA). This important observation brings the investigators to believe that the current priming question’s wording may not be able to fully distinguish between the PNA and ONA groups. Further investigation is necessary to further validate the use of the priming question to distinguish between all three groups.

One of the variations from the original DRAW tool was to provide a scaled answer choice, instead of presenting a binary choice of ‘yes’ or ‘no’. A Likert-scaled response allowed for more detailed response from the patients. Interestingly, six questions had an average score greater than 2 within the intervention group, and all questions had higher average scores within the intervention group compared to the control group (Table 5). The study investigators believed that providing the scaled answer choices allowed participants to provide an accurate reflection of their own medication-taking behavior.

The M-DRAW checklist and GUIDE strategy provided recommendations to clinicians on how to systematically approach each identified barrier. Using the M-DRAW tool, the investigators were able to ask appropriate follow-up questions to identify the root cause contributing to the medication non-adherence, and provide a tailored consultation. In the intervention group, more patients (22.2%) reported feeling uncomfortable taking medication(s) while with family or friends, compared to no subjects (*n* = 0) in the control group. One participant reported feeling ashamed taking out a syringe to prepare a dose of insulin injection anywhere outside of the house, which contributed to missing a dose or two now and then during family-gathering events. The M-DRAW tool could be successful in identifying signals such as these that would enable the pharmacist to ask tailored follow-up questions to investigate any issues contributing to medication non-adherence. Identifying these barriers would help healthcare providers initiate a conversation about specific issues to determine individualized, patient-centered solutions to adherence issues.

The study findings should be interpreted with caution because of several limitations. First, the small sample size (*n* = 26) and convenience sampling limits generalizability of the results. Second, test–retest reliability was not performed. The study period lasted for a shorter duration than expected, and follow-up assessments were not collected as planned at the initial stage of the study protocol development. Future research will focus on testing the M-DRAW tool for construct validity, examining the impact of the pharmacist using the M-DRAW tool to identify patients with medication adherence issues and choosing a patient-tailored intervention. The impact of the pharmacist-initiated intervention on patient outcomes and medication adherence also can be evaluated.

## 5. Conclusions

The use of the M-DRAW tool showed acceptable reliability to identify barriers to medication adherence. More barriers were identified within the patients with unintentional non-adherence compared to those who adhere to the medication. The priming question assessing the self-reported adherence level showed good discriminant validity.

## Figures and Tables

**Table 1:** Comparison of currently available tools to assess factors contributing to medication non-adherence [5,6,7].

	Tools	MMAS-8	BMQ	ABQ	DRAW	M-DRAW
Domain (Description)						
Patient-related	Lack of belief in benefit of treatment Lack of insight into the illness Belief in medication and complementary and alternative medicine Trust in health care professionals	√	√		√	√
Therapy-related	Stigma Treatment interfering with lifestyle Complexity of treatment Number of medications			√	√	√
Condition-related	Chronic AsymptomaticMental health conditions Number of comorbid conditions Duration of disease Presence of depressive symptom	√				√
Socioeconomic	Gender Education Functional health literacy level Social support Competing priority Health insurance Access to medication Cost of medication				√	√

MMAS-8, 8-item Morisky Medication Adherence Scale; BMQ, Brief Medication Questionnaires; ABQ, Adherence Barriers Questionnaires; DRAW, Drug Adherence Work-up; M-DRAW, Modified Drug Adherence Work-up.

**Table 2:** Self-reported adherence level categorized by answers to the priming question.

**Priming Question:** You have been prescribed medication(s) for your health condition(s) which is to be taken regularly. How would you describe your past experience with taking your medication(s)?	
**Answer Choices**	**Adherence Level**
(a) I want to be very regular in taking my medication(s), but I am not always good with it due to some challenges.	Partial non-adherence (PNA or unintentional non-adherence)
(b) I take my medication(s) regularly (9 out of 10 times)	Adherence
(c) I am not very regular in taking my medication(s) because I feel unwilling.	Outright non-adherence (ONA or intentional non-adherence)

**Table 3:** Demographic and health-related characteristics of study participants.

	Control (*n* = 17)	Intervention (*n* = 9)	*p*-Value
Age, mean year ± SD	59.1 ± 6.9	56.0 ± 11.0	0.46
Gender, *n* (%)			0.61
Female	9 (52.9)	5 (55.6)	
Male	8 (47.1)	4 (44.4)	
Ethnicity, *n* (%)			0.53
White	6 (37.5)	4 (44.4)	
Hispanic/Latino	7 (43.8)	3 (33.3)	
Black	0 (0.0)	1 (11.1)	
Asian/Pacific Islander	3 (18.8)	1 (11.1)	
Annual income, *n* (%)			0.47
Less than $25,000	6 (40.0)	3 (33.3)	
$25,000 or more	9 (60.0)	6 (66.6)	
Highest education, *n* (%)			0.71
High school or some college	11 (68.6)	5 (55.5)	
Bachelor’s degree or above	5 (31.6)	4 (44.4)	
Present health condition(s), *n* (%)			
Hypertension	14 (82.4)	8 (88.9)	0.66
Dyslipidemia	11 (64.7)	4 (44.4)	0.32
Diabetes	9 (52.9)	4 (44.4)	0.68
Chronic pain	5 (29.4)	2 (22.2)	0.69
Have prescription coverage *n* (%)			
Yes	17 (100.0)	7 (77.8)	0.04

SD, Standard Deviation.

**Table 4:** Baseline number of adherence barriers, adherence rate and clinical measures.

		Control	Intervention	*p*-Value
Number of barriers identified		1.2 ± 1.3	4.4 ± 2.5	0.004
mean ± SD		(*n* = 17)	(*n* = 9)	
Adherence rate	PDC ^1^	80.4 ± 10.3	77.1 ± 21.0	0.687
mean ± SD		(*n* = 12)	(*n* = 6)	
Clinical baseline measures ^2^	A1C	7.8 ± 1.3	6.4 ± 1.1	0.161
mean ± SD		(*n* = 8)	(*n* = 3)	
	Blood pressure	137/78	138/87	
		(*n* = 13)	(*n* = 8)	
	Total cholesterol	193 ± 37.7	152 ± 45.3	0.262
		(*n* = 6)	(*n* = 3)	
	HDL	44.3 ± 20.0	36.3 ± 11.7	0.477
		(*n* = 6)	(*n* = 3)	

SD, standard deviation; PDC, proportion of days covered; HDL, high-density lipoprotein; ^1^ Excluded those with less than 2 fill dates; ^2^ excluded those without the clinical measures from the electronic medical records (EMRs).

**Table 5:** Responses to the M-DRAW checklist (Number of barriers identified in study groups).

	Checklist Item	Control (*n* = 17)	Intervention (*n* = 9)	*p*-Value (2-Sided)
1	Do you feel unsure about how/when to take your medications?	1.4	1.7	0.337
2	Do you have any difficulty getting your medications on time from the pharmacy?	1.5	1.7	0.611
3	Do you have difficulty keeping track of all your medication schedules throughout the day?	1.2	2.2	0.060
4	Do your medications give you side effects that make you NOT want to take it?	1.5	2.7	0.004 *
5	Do you worry about what foods or other medications might interact with your medication?	1.4	2.1	0.099
6	Do you feel that you can take more or less of your medication than the prescribed dose to fit your lifestyle?	1.2	2.3	0.031 *
7	Do you feel like you don’t get any benefits from taking your medication?	1.2	1.8	0.151
8	Do you feel uncomfortable about taking your medication while you are out with family and friends?	1.1	1.7	0.099
9	Do you consider it a burden that you have to take your medications for the rest of your life?	1.9	2.4	0.228
10	Do you have doubts about whether your health condition needs to be treated?	1.2	2.0	0.130
11	Do you have doubts if taking your medication will improve your health condition in the long term?	1.5	1.7	0.699
12	Do you feel that you are NOT receiving the best possible treatment available from your health care provider?	1.4	1.6	0.486
13	Do you have any other doubts or concerns about taking your medication? ^1^	N/A	N/A	N/A

* Statistically significant difference with equal variances not assumed; ^1^ open-ended question item.

## Supplementary Materials

The following are available online at http://www.mdpi.com/2226-4787/5/3/52/s1, Figure S1: Modified Drug Adherence Work-up Tool (M-DRAW); Figure S2: Demographic Information.

## References

[1] Iuga A.O., McGuire M.J. (2014). Adherence and health care costs. Risk Manag. Healthc. Policy.

[2] Clifford S., Barber N., Horne R. (2008). Understanding different beliefs held by adherers, unintentional nonadherers, and intentional nonadherers: Application of the Necessity-Concerns Framework. J. Psychosom. Res..

[3] Lehane E., McCarthy G. (2007). Intentional and unintentional medication non-adherence: A comprehensive framework for clinical research and practice? A discussion paper. Int. J. Nurs. Stud..

[4] Costello K., Kennedy P., Scanzillo J. (2008). Recognizing nonadherence in patients with multiple sclerosis and maintaining treatment adherence in the long term. Medscape J. Med..

[5] Svarstad B.L., Chewning B.A., Sleath B.L., Claesson C. (1999). The Brief Medication Questionnaire: A tool for screening patient adherence and barriers to adherence. Patient Educ. Couns..

[6] Muller S., Kohlmann T., Wilke T. (2015). Validation of the Adherence Barriers Questionnaire—An instrument for identifying potential risk factors associated with medication-related non-adherence. BMC Health Serv. Res..

[7] Doucette W.R., Farris K.B., Youland K.M., Newland B.A., Egerton S.J., Barnes J.M. (2012). Development of the Drug Adherence Work-up (DRAW) tool. J. Am. Pharm. Assoc..

[8] Osterberg L., Blaschke T. (2005). Adherence to medication. N. Engl. J. Med..

